# Characterization of orthotopic xenograft tumor of glioma stem cells (GSCs) on MRI, PET and immunohistochemical staining

**DOI:** 10.3389/fonc.2022.1085015

**Published:** 2022-12-15

**Authors:** Yufei Liu, Wenzhen Zhu, Hongquan Zhu, Jiaxuan Zhang, Ju Zhang, Nanxi Shen, Jingjing Jiang, Yunjing Xue, Rifeng Jiang

**Affiliations:** ^1^ Department of Radiology, Tongji Hospital, Tongji Medical College, Huazhong University of Science and Technology, Wuhan, Hubei, China; ^2^ Department of Radiology, Fujian Medical University Union Hospital, Fuzhou, Fujian, China

**Keywords:** glioma stem cell, nude rat, magnetic resonance imaging, positron emission tomography, immunohistochemistry

## Abstract

**Introduction:**

The orthotopic xenograft tumors of human glioma stem cells (GSCs) is a recent glioma model with genotype and phenotypic characteristics close to human gliomas. This study aimed to explore the imaging and immunohistochemical characteristics of GSCs gliomas.

**Methods:**

The rats underwent MRI and ^18^F-FDG PET scan in 6^th^–8^th^ weeks after GSCs implantation. The MRI morphologic, DWI and PET features of the tumor lesions were assessed. In addition, the immunohistochemical features of the tumor tissues were further analyzed.

**Results:**

Twenty-five tumor lesions were identified in 20 tumor-bearing rats. On structural MRI, the average tumor size was 30.04±17.31mm^2^, and the intensity was inhomogeneous in 76.00% (19/25) of the lesions. The proportion of the lesions mainly presented as solid, cystic and patchy tumor were 60.00% (15/25), 16.00% (4/25) and 24.00% (6/25), respectively. The boundary was unclear in 88.00% (22/25), and peritumoral mass effect was observed in 92.00% (23/25) of the lesions. On DWI, 80.00% (20/25) of the lesions showed increased intensity. Of the 14 lesions in the 11 rats underwent PET scan, 57.14% (8/14) showed increased FDG uptake. On immunohistochemical staining, the expression of Ki-67 was strong in all the lesions (51.67%±11.82%). Positive EGFR and VEGF expression were observed in 64.71% (11/17) and 52.94% (9/17) of the rats, whereas MGMT and HIF-1α showed negative expression in all the lesions.

**Discussion:**

GSC gliomas showed significant heterogeneity and invasiveness on imaging, and exhibited strong expression of Ki-67, partial expression of EGFR and VEGF, and weak expression of MGMT and HIF-1α on immunohistochemical staining.

## 1 Introduction

Many murine glioma models fail to accurately mimic the high heterogeneity and invasiveness of human gliomas, which hinder the development of effective therapeutics and imaging agents for gliomas (1–3). For example, the U87, U251 and U373 human glioma cell lines grew in the brain of mice mainly exhibit as enlargement of the tumor but invasive growth (1, 4–7). Although these models are still widely used in many longitudinal studies, they have many limitations (8, 9).

The need for a new glioma model has led to the transplantation of human glioma stem cells (GSC), a subpopulation of glioma stem cells in glioblastoma that are responsible for tumor initiation, treatment resistance and recurrence (10), into the brain of immuno-compromised mice (11). Studies have shown that this transplanted tumor has the genotype and phenotypic characteristics very close to human gliomas (7). Therefore, it has great potential to serve as a more reliable and representative model of glioma than many commonly utilized cancer cell lines.

Magnetic resonance imaging (MRI) is the imaging modality of choice to monitor patient with gliomas and treatment effects, and has also been widely applied to rat or murine models of glioma. MRI is able to non-invasively display the growth and volume of tumors in the brain of a living rat. Recently, in addition to structural MRI, diffusion-weighted imaging (DWI) and ^18^F-2-fluro-D-deoxy-glucose (^18^F-FDG) positron emission computed tomography (PET) have also been widely used in the evaluation of brain tumors, and these methods have shown great value in the evaluation of tumor grades, cellular proliferation and therapeutic response (12). Previous studies have demonstrated that MRI provides detailed structural images for orthotopic xenograft gliomas (13–15). However, few studies have discussed the characteristics of DWI and glucose metabolic imaging for the transplanted gliomas.

On the other hand, molecular studies have provided important new insights into the complex genetic, chromosomal, and epigenetic changes within gliomas that accompany glioma formation and maintenance. Evidence has shown that several molecules such as Ki-67, epidermal growth factor receptor (EGFR), vascular endothelial growth factor (VEGF), O-6-methylguanine-DNA methyltransferase (MGMT), and hypoxia-inducible factor 1-alpha (HIF-1α) play important roles in the growth of glioma (16–18). However, the characteristics of those molecules in the GSC transplanted gliomas are still not clear up to now.

It is clear that a truly representative glioma model needs to be combined with the structural MRI, DWI, metabolic images and molecular profiles to become an excellent testing platform. Therefore, this study attempted to describe the MRI, PET and molecular characteristics of an orthotopic xenograft tumor of GSCs, in order to improve the glioma platform for preclinical testing of new therapeutic and imaging approaches.

## 2 Materials and methods

### 2.1 Animals and implantation of tumor cells

A human glioblastoma cell line (called NCH421k) was established from resected tumors, passaged serially as tumor xenografts, and cultured in DMEM/F12 medium supplemented with 20% BIT 9500, bFGF and EGF. Cultures were incubated in a controlled atmosphere at 37°C with 5% CO_2_ and 20%O_2_. The NCH421k cells (10uL) were stereotactically implanted in the nude rats’ caudate putamen. The nude rats were first anesthetized and placed in a stereotactic frame. A 2-cm scalp incision was made on the right side of the skull, 1 mm posterior to the coronal suture, 3 mm right from the midline, and 5 mm deep from the surface at the bregma level, to allow the passage of a Eppendorf syringe to inject 10 ul cell suspension carrying a total of 1,000,000 NCH421k cells (19). The nude rats weighed between 180 to 340 g. All animal experiments were approved by the Animal Research Committee of the Tongji Medical College of Huazhong University of Science and Technology and were performed according to the guidelines of the Division of Laboratory Animal Medicine at the Tongji Medical College.

### 2.2 MRI data acquisition

The nude rats underwent MRI scanning in the 6^th^-8^th^ week post-tumor implantation. The diameter of the tumors was approximately 4-5 mm. Imaging experiments were performed on a 3.0 T MR system (Discovery LS MR 750, GE) with a rat-coil (CG-MUC30-H300-AG, Chenguang, Shanghai, China) of 50 mm for signal reception.

*In vivo* multi-slice images of rat brains were acquired in the coronal plane. All the MRI sequence were having the same slice thickness of 2.0 mm, slice number of 12 slices and field of view of 50 × 50 mm^2^, which would facilitate the further ROI placement. T2-weighted image (T2WI) was acquired using a fast spin echo (FSE) sequence and the following parameters: repetition time (TR) = 3.5s; echo time (TE) = 99 ms; matrix = 256 × 256; and resolution = 0.2 × 0.2 mm^2^. Three-dimensional T1-weighted image (T1WI) was acquired using a three-dimensional fast spoiled gradient-recalled (3D-FSPGR) sequence and the following parameters: repetition time (TR) = 6.3 ms; echo time (TE) = 3.2 ms; inversion time = 390ms, flip angle = 12°, matrix for acquisition = 128 × 128, matrix for reconstruction = 256 × 256, and resolution = 0.2 × 0.2 mm^2^. DWI image was acquired using a multiple-slice, spin echo-echo planar imaging sequence (TR = 2 s, TE = 83.4 ms, matrix for acquisition = 96 × 64, matrix for reconstruction = 256 × 256, resolution = 0.2 × 0.2 mm^2^, number of average = 12).

### 2.3 PET data acquisition

The nude rats fasted for at least 6 hours before PET imaging. Image acquisition started at 50 min after a bolus injection of 18.5MBq/kg of ^18^F-FDG for each rats using a small-animal PET scanner (Raycan Technology Co., Ltd, Suzhou, China). The whole brain PET scan included only one bed position and was acquired in a three-dimensional imaging mode (field of view = 140 × 140 mm^2^, resolution = 0.5 × 0.5 × 0.5 mm^3^), and the acquisition time for the bed was 15 min.

### 2.4 Data processing

Apparent diffusion coefficient (ADC) map was calculated using ImageJ (Version 1.49b, NIH) by fitting the b_0_ image and DWI images (b = 1000 s/mm^2^) into the mono-exponential equation: S_b_/S_0_ = exp (–b × ADC), where S_b_ is the diffusion-weighted signal intensity of the voxels for the b value, and S_0_ is the signal intensity of the corresponding voxels for the b_0_ image.

The PET images were manually registered to the T2-FSE using Amide (version: 0.9.0, http://amide.sourceforge.net/). Maximum standard uptake value (SUV_max_) map was calculated as follows: SUV_max_ = maximum voxel activity/(injected dose/body weight) (20).

### 2.5 Image analysis

Image data were reviewed and assessed by two radiologists (with 13 and 8 years of experience respectively) and a nuclear medicine physician (with 8 years of experience) who were blinded to the results of the other observers.

The MRI morphologic features of the tumor lesions were evaluated, including lesion number, size, intensity, boundary, and peritumoral mass effect. The lesion size was presented as area of the lesion in the maximal tumor slice measured using ImageJ (Version 1.49b, NIH). The DWI intensity and FDG uptake of the tumor lesions were defined as normal or decreased if they were equal to or lower than those of the surrounding brain tissue respectively, otherwise the DWI intensity and FDG uptake of the tumor lesions were increased.

Regions of interest (ROI) over the solid region of the tumor and contralateral pons were manually delineated on coronal T2-FSE. Identifiable cystic components, necrosis, hemorrhage and calcification were avoided in delineation of the solid region of the tumor. The ROIs were transferred from T2-FSE to ADC and SUV maps (21). Because there may be a slight deformation between the diffusion-imaging image and the T2-FSE image, the ROI needs to be manually adjusted before measurement.

Average ADC value and SUV_max_ were calculated for each ROI. The values in the solid region of the tumor were normalized to the corresponding mean values in the contralateral pons of each rat to eliminate whole-brain variations between individuals. Therefore, normalized apparent diffusion coefficient (nADC) and normalized maximum SUV (nSUV_max_) values of the tumor lesions were calculated.

### 2.6 Histopathological evaluation

The brains of the nude rats were removed and sliced to 2 mm thickness, matching the MR slice thickness. The 2 mm slices were fixed in formalin, sectioned to 5 μm, and stained with hematoxylin and eosin (H&E). The immunohistochemical staining was further performed for solid tumor region using the Envision method. For Ki-67, the Ki-67 labeling index (Ki-67 LI) was measured, which is defined as the percentage of positive cells in the highest density of the stained areas. For EGFR, VEGF, MGMT, and HIF-1α, staining was interpreted as positive when 5% or more tumor cells showed a strong nucleus or cytoplasmic staining, whereas staining of less than 5% of tumor cells was counted as negative findings.

### 2.7 Statistical analysis

Statistical analyses were performed with SPSS software (version 20, IBM, Armonk, NY, USA). The tumorigenesis rate of the glioma model was first calculated. For tumor-bearing animals, descriptive statistics of the tumor lesion features under MRI, PET and immunohistochemistry was performed, including the lesion number, size, intensity, boundary, peritumoral mass effect, DWI intensity, FDG uptake, Ki-67, EGFR, VEGF, MGMT, and HIF-1α expression of the tumor. Spearman correlation analysis was further performed to evaluate the relationship between mean nADC and nSUV_max_ in the tumor area and between Ki-67 LI and image features. Mann-Whitney U test or Chi-squared test was used to compare the difference in image features between different levels of EGFR and VEGF expression.

## 3 Results

### 3.1 Rats

**Figure 1:**
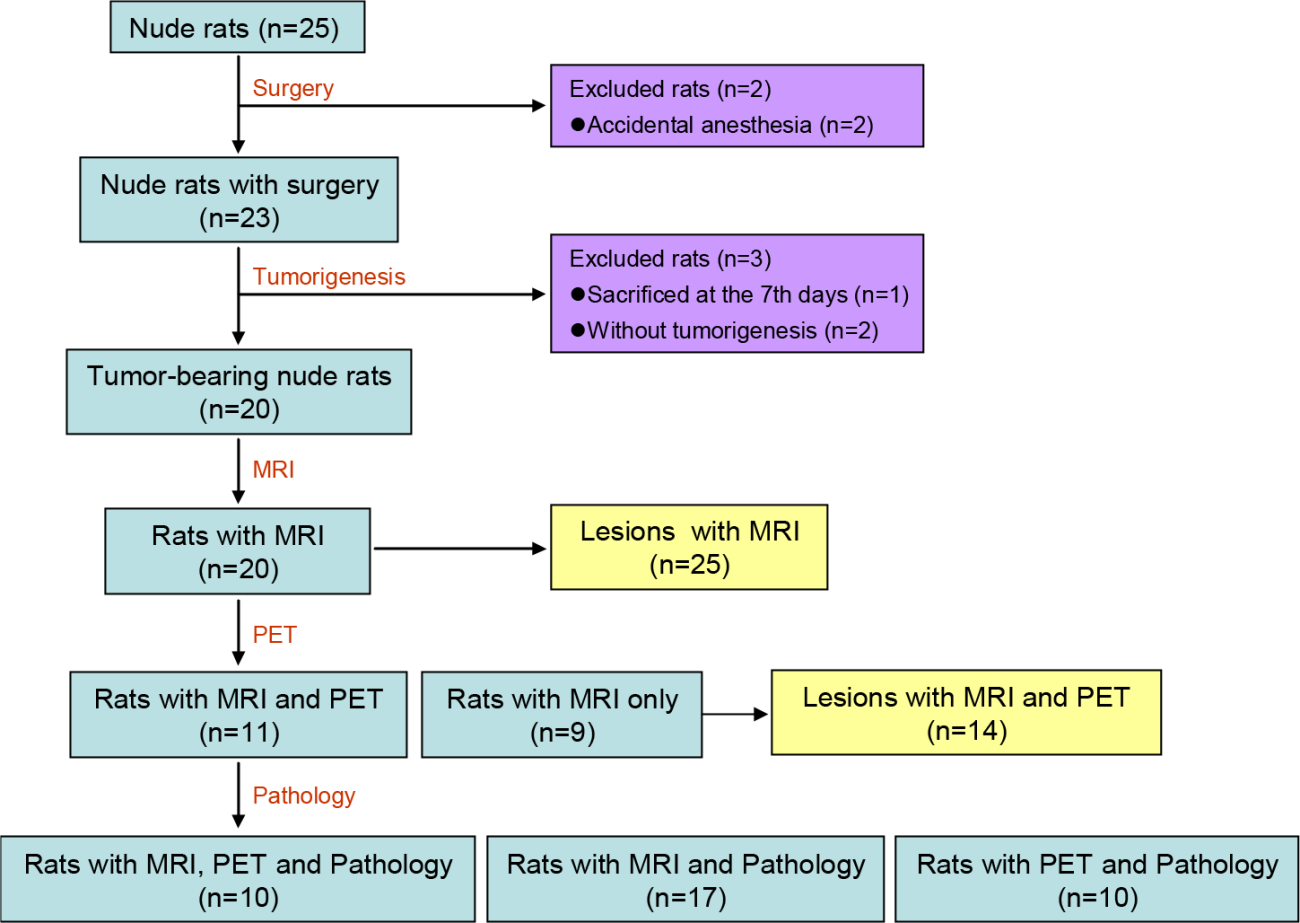
Flow Chart of the included nude rats and tumor lesions. MRI, magnetic resonance imaging; PET, positron emission computed tomography.

Of the 25 nude rats, 2 were sacrificed at the time of surgery due to an accidental anesthesia, and another one was sacrificed 7 days after the surgery. Of the remaining 22 nude rats, there were 20 nude rats verified to bear gliomas under MRI at 6-8 weeks after the surgery. The tumorigenesis rate was 90.91% (20/22). Based on the MRI of these 20 tumor-bearing rats, 75.00% (15/20) rats bore single tumor lesion, whereas the other 25.00% (5/20) rats bore multiple tumor lesions, and a total of 25 tumor lesions were identified. The 55.00% (11/20) rats carrying a total of 14 tumor lesions underwent additional PET scan. Finally, the brain tissue of 17 nude rats was preserved and the pathological and immunohistochemical analysis were successfully carried out. The flow chart of the included nude rats and tumor lesions is shown in **Figure 1**.

### 3.2 Imaging features

**Figure 2:**
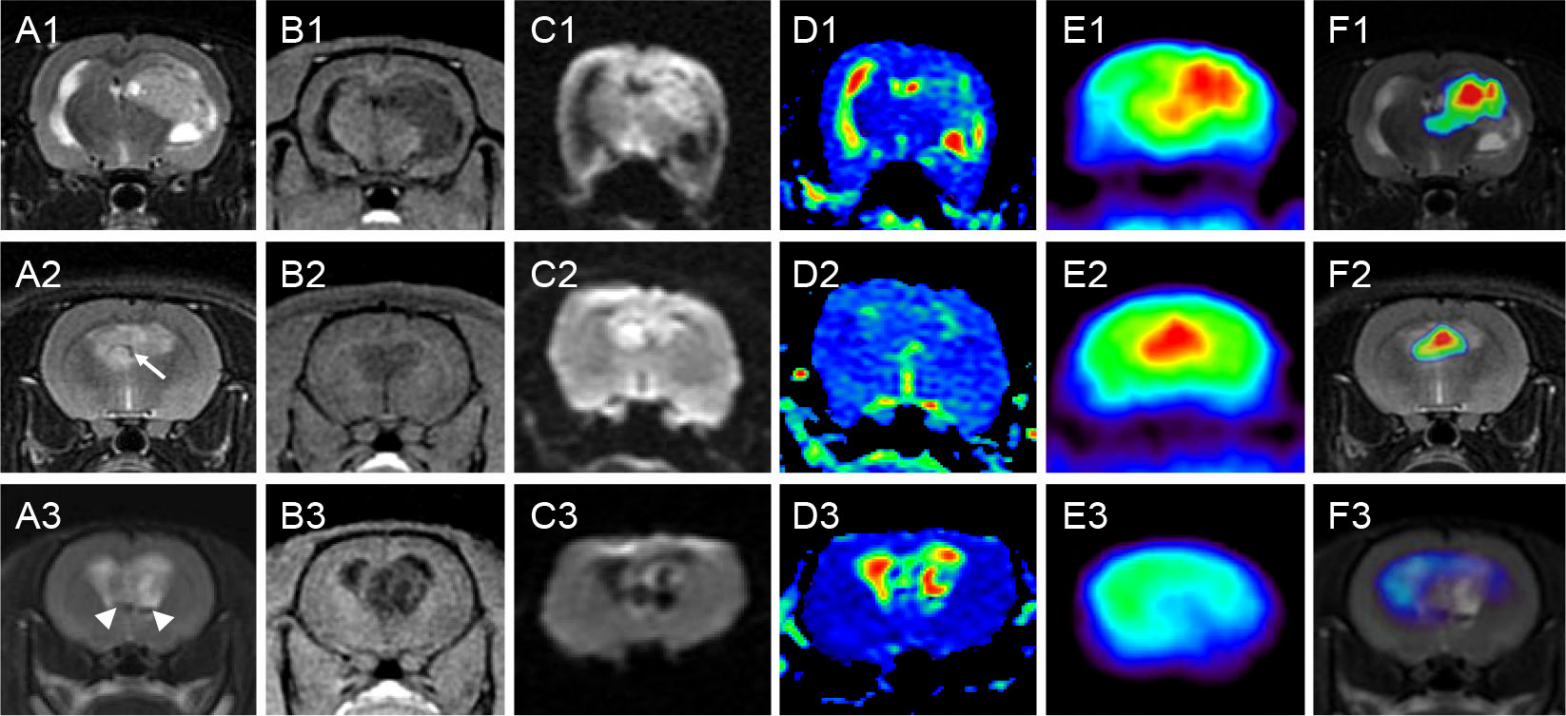
Characteristics of representative GSC tumors on MRI and PET. Rows 1-3 correspond to three nude rats with orthotopic xenograft tumors of GSCs. Columns **(A–F)** are coronal T2WI, 3D-FSPGR, DWI, ADC, PET and fusion map of T2WI and PET, respectively. All the three lesions presented low intensity on T1WI and high intensity on T2WI, and the intensity was inhomogeneous. Lesion in rat 1 was mainly presented as a solid tumor with cystic degeneration. Lesion in rat 2 was mainly presented as a patchy tumor with vasculature in the tumor (arrow). Lesion in rat 3 was mainly presented as a cystic tumor with component with very low intensity (arrowhead) on T2WI indicating remote hemorrhage. The boundaries were unclear and different levels of mass effect were found in all the three lesions. DWI images showed increased intensity in the solid or patchy tumor lesions (rat 1 and 2), but displayed decreased intensity in the regions of cystic degeneration (rat 3). PET also showed high uptake of ^18^F-FDG in the solid or patchy tumor regions (rat 1 and 2), but showed low uptake in the cystic tumor (rat 3). GSC, glioma stem cell; MRI, magnetic resonance imaging; PET, positron emission computed tomography; T2WI, T2-weighted image; 3D-FSPGR, three-dimensional fast spoiled gradient-recalled sequence; DWI, diffusion weighted imaging; ADC, apparent diffusion coefficient; T1WI, T1-weighted image; FDG, fluoro-2-deoxy-D-glucose.

Characteristics of the GSCs tumor lesions based on MRI and PET are listed in **Table 1**, and the representative lesions are shown in **Figure 2**. On structural MRI, the average area of the whole tumor in the maximum slice for the 25 tumor lesions was 30.04 ± 17.31mm^2^, and the average area of the solid tumor in the maximum slice was 20.68 ± 15.30mm^2^. All lesions, 100.00% (25/25), presented low intensity on T1WI and high intensity on T2WI, and the intensity of 76.00% (19/25) lesions was inhomogeneous. There were 68.00% (17/25) lesions containing cystic degeneration or necrosis, 32.00% (8/25) lesions containing low intensity on T2WI indicating remote hemorrhage, and 52.00% (13/25) lesions containing vasculature with low intensity due to flowing avoid effect in or around the lesion. Of the tumor lesions, 16.00% (4/25) were mainly presented as cystic tumors, 60.00% (15/25) were mainly presented as solid tumors, whereas the other 24.00% (6/25) were mainly presented as patchy tumors. There were 88.00% (22/25) lesions showed unclear boundary, 48.00% (12/25) lesions accompanied with peritumoral edema and 92.00% (23/25) lesions accompanied with mass effect of varying degrees surrounding the tumor lesions presenting as compressed ipsilateral brain parenchyma and ventricle and the shifted midline to the contralateral side. On DWI, 80.00% (20/25) lesions showed increased intensity, and the other 20.00% (5/25) lesions showed normal or decreased intensity. On PET, 57.14% (8/14) tumor lesions showed increased FDG uptake, and the other 42.86% (6/14) tumors showed normal or decreased FDG uptake.

**Table 1:** Characteristics of tumor lesions on MRI and PET.

Characteristics on MRI and PET	Number/Mean ± SD/Percent
Number of tumor-bearing nude rats	20
Number of tumor lesions	25
Size on MRI	
Tumor size in the maximum slice (mm^2^)	30.04 ± 17.31
Solid tumor size in the maximum slice (mm^2^)	20.68 ± 15.30
Intensity on MRI	
Low intensity on T1WI and high on T2WI	100.00% (25/25)
Inhomogeneous	76.00% (19/25)
Homogeneous	24.00% (6/25)
Containing cystic degeneration or necrosis	68.00% (17/25)
Containing remote hemorrhage	32.00% (8/25)
Containing vasculature in or around the tumor	52.00% (13/25)
Mainly presented as cystic tumor	16.00% (4/25)
Mainly presented as solid tumor	60.00% (15/25)
Mainly presented as patchy tumor	24.00% (6/25)
Boundary on MRI	
Unclear	88.00% (22/25)
Clear	12.00% (3/25)
Edema on MRI	
Yes	48.00% (12/25)
No	52.00% (13/25)
Mass effect on MRI	
Yes	92.00% (23/25)
No	8.00% (2/25)
Intensity on DWI	
Increased	80.00% (20/25)
Normal or decreased	20.00% (5/25)
^18^F-FDG uptake	
Increased	57.14% (8/14)
Normal or decreased	42.86% (6/14)

MRI, magnetic resonance imaging; PET, positron emission computed tomography; DWI, diffusion weighted imaging; FDG, fluoro-2-deoxy-D-glucose.

**Figure 3:**
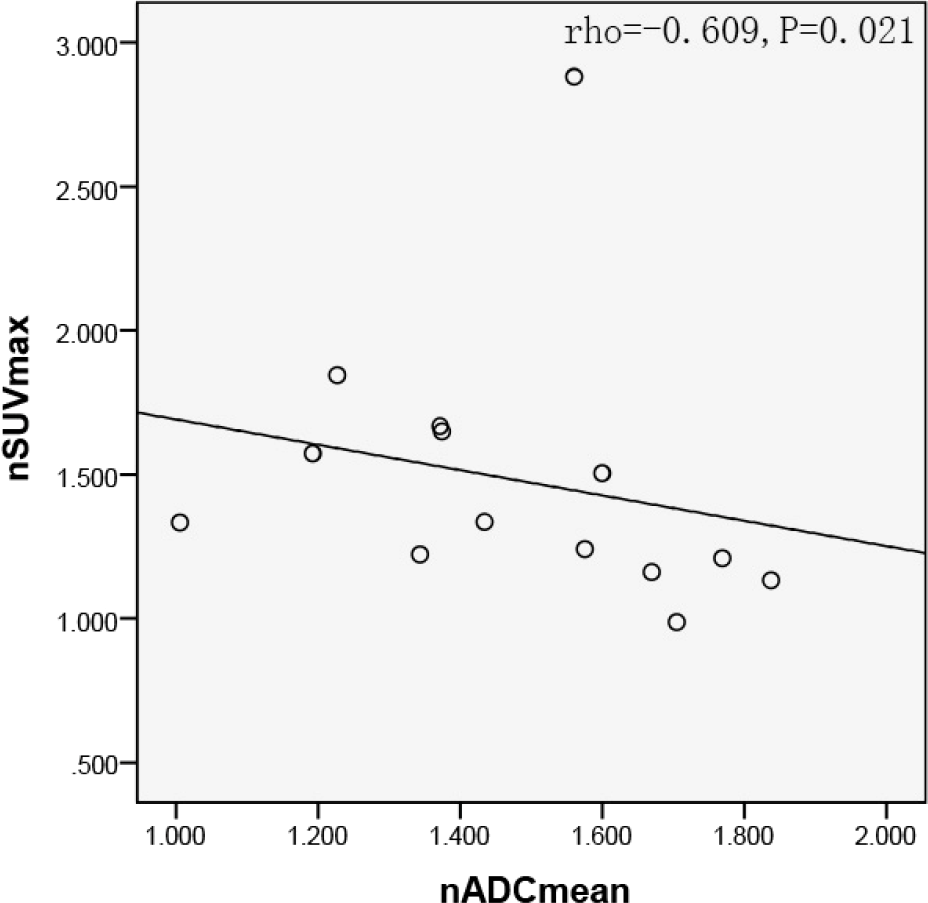
Scatter diagram of nSUV_max_ with average nADC. Significant negative correlation were found between nSUV_max_ and nADC in the solid tumor region (rho=-0.609, P=0.021). SUV, standard uptake value; ADC, apparent diffusion coefficient.

In quantitative ROI analysis, 25 ROIs over the solid region of the tumor and 20 ROIs over the contralateral pon were drawn. The average size of ROIs over the solid region of the tumor was 19.20 ± 11.01 mm^2^, whereas ROIs with fixed size of 2.72 mm^2^ were drawn over the contralateral pons. The nADC in the solid tumor area was 1.466 ± 0.260, and the nSUV_max_ of the 14 tumor lesions was 1.482 ± 0.470. The Spearman correlation analysis further showed that there was a significant correlation between the nSUV_max_ and the nADC for the 14 tumor lesions (rho=-0.609, P=0.021). The corresponding scatter diagram is exhibited in **Figure 3**.

### 3.3 Immunohistochemical features

**Figure 4:**
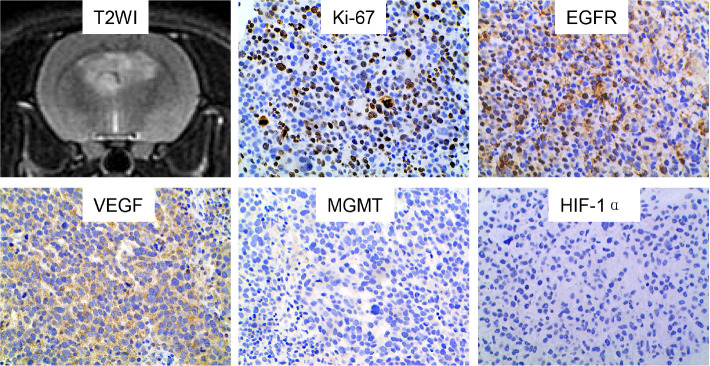
Characteristics of a representative GSC tumor on immunohistochemical staining. The tumor lesion showed positive expression of Ki-67, EGFR and VEGF. In contrast, negative expression was found for MGMT and HIF-1α. GSC, glioma stem cell; T2WI, T2-weighted image; EGFR, epidermal growth factor receptor; VEGF, vascular endothelial growth factor; MGMT, O-6-methylguanine-DNA methyltransferase; HIF-1α, hypoxia-inducible factor 1-alpha.

For rats with multiple tumor lesions, a representative lesion was selected for the further pathological and immunohistochemical analysis. The results of the immunohistochemical analysis of the 17 tumor lesions are exhibited in **Table 2**, and the immunohistochemical features of a representative lesion are exhibited in **Figure 4**. All the tumor lesions showed strong expression of Ki-67, and the mean and standard deviation of Ki-67 LI were 51.67% ± 11.82%. Positive EGFR expression of the tumor was found in 64.71% (11/17) nude rats. Similarly, positive VEGF expression of the tumor was observed in 52.94% (9/17) nude rats. In contrast, both MGMT and HIF-1α had negative expression in all the tumor lesions of the 17 nude rats.

**Table 2:** Characteristics of tumor lesions on immunohistochemical staining.

Characteristics on immunohistochemical staining	Number/Mean ± SD/Percent
Number of the total rats	17
Ki-67 LI (%)	51.67 ± 11.82
Positive (≧5%)	100.00% (17/17)
Negative (<5%)	0.00% (0/17)
EGFR	
Positive (≧5%)	64.71% (11/17)
Negative (<5%)	35.29% (6/17)
VEGF	
Positive (≧5%)	52.94% (9/17)
Negative (<5%)	47.06% (8/17)
MGMT	
Positive (≧5%)	0.00% (0/17)
Negative (<5%)	100.00% (17/17)
HIF-1α	
Positive (≧5%)	0.00% (0/17)
Negative (<5%)	100.00% (17/17)

EGFR, epidermal growth factor receptor; VEGF, vascular endothelial growth factor; MGMT, O-6-methylguanine-DNA methyltransferase; HIF-1α, hypoxia-inducible factor 1-alpha.

### 3.4 Association of image features with immunohistochemical features

For Ki-67, Spearman correlation analysis of Ki-67 LI indicated that there was lack of significant correlation between Ki-67 LI and tumor size (rho=0.400, p=0.112), solid tumor size (rho=0.414, p=0.098) or nADC_mean_ (rho=0.086, p=0.743) for the 17 tumor-bearing nude rats. Similarly, no significant correlation was observed between Ki-67 LI and nSUV_max_ (rho=0.115, p=0.751) for the 10 tumor-bearing nude rats.

For EGFR and VEGF, Mann-Whitney U test was used to compare the difference in imaging features between different levels of EGFR and VEGF expression, and the results are shown in **Table 3**. It was observed that solid tumor size was significantly larger in tumors with positive EGFR expression than those with negative EGFR expression (Z=-2.312, p=0.021). In contrast, no significant difference was observed for the other imaging features between different levels of EGFR expression. Tumor with positive VEGF expression also showed larger size compared with those with negative VEGF expression, but without reaching significance (Z=-1.828, p=0.068). Similarly, tumor with positive VEGF expression seemed to have higher probability to contain vasculature in or around the tumor (p=0.057). In contrast, no significant difference was observed for the other imaging features between different levels of VEGF expression.

**Table 3:** Association of imaging features with levels of EGFR and VEGF expression.

Imaging features	EGFR				VEGF			
	Positive(n=11)	Negative(n=6)	Z/χ²	*p*	Positive(n=9)	Negative(n=8)	Z/χ²	*p*
Size								
Tumor size in the maximum slice (mm^2^)	27.11 ± 11.26	24.30 ± 7.16	-0.804	0.421	29.46 ± 9.32	23.07 ± 10.38	-1.540	0.124
Solid tumor size in the maximum slice (mm^2^)	23.27 ± 12.31	8.24 ± 3.99	-2.312	0.021*	21.20 ± 12.86	16.32 ± 13.18	-1.828	0.068
Intensity								
Inhomogeneous/homogeneous	8/3	5/1	NA	1.000	7/2	6/2	NA	1.000
Containing cystic degeneration or necrosis (Yes/No)	6/5	5/1	NA	0.333	5/4	6/2	NA	0.620
Containing remote hemorrhage (Yes/No)	4/7	3/3	NA	0.644	3/6	4/4	NA	0.637
Containing vascular in or around the tumor (Yes/No)	7/4	2/4	NA	0.335	7/2	2/6	NA	0.057
Mainly presented as cystic tumor (Yes/No)	1/10	3/3	NA	0.099	2/7	2/6	NA	1.000
Mainly presented as solid tumor (Yes/No)	7/4	2/4	NA	0.335	5/4	4/4	NA	1.000
Mainly presented as patchy tumor (Yes/No)	3/8	1/5	NA	1.000	2/7	2/6	NA	1.000
Boundary (unclear/clear)	10/1	6/0	NA	1.000	9/0	7/1	NA	0.471
Edema (Yes/No)	5/6	3/3	NA	1.000	4/5	4/4	NA	1.000
Mass effect (Yes/No)	10/1	5/1	NA	1.000	8/1	7/1	NA	1.000
nADC_mean_	1.50 ± 0.25	1.49 ± 1.88	-0.302	0.763	1.57 ± 0.21	1.44 ± 0.24	-0.674	0.501
nSUV_max_	1.65 ± 0.60 (n=7)	1.18 ± 0.18 (n=3)	-1.254	0.210	1.63 ± 0.77 (n=5)	1.39 ± 0.23 (n=5)	-0.104	0.917

That * indicates p<0.05. NA, not applicable. ADC, apparent diffusion coefficient; SUV, standard uptake value; EGFR, epidermal growth factor receptor; VEGF, vascular endothelial growth factor.

As both MGMT and HIF-1α showed negative expression in all the tumor lesions, correlation analysis or difference comparison was not further performed.

## 4 Discussion

GSCs gliomas have shown highly heterogeneity and invasiveness very close to human gliomas, indicating that it has great potential to serve as a representative glioma model (7).

At present, structural MRI has been increasingly used to assess drug effects in animal models of brain cancer (1, 22–24), including orthotopic xenograft tumor of GSCs (13–15). However, few studies have explored the DWI and glucose metabolism of GSCs gliomas. Therefore, in addition to structural MRI, this study further evaluated the diffusion MRI and glucose metabolism characteristics of the GSC glioma, aiming to improve the test platform for glioma.

On structural MRI, the GSC glioma showed significant heterogeneity in this study. The intensity of all the tumor lesions was low on T1WI and high on T2WI, however, most of the lesions had inhomogeneous intensity. The tumor lesions were more likely to present as solid tumors, followed by patchy and cystic tumors. Cystic degeneration, necrosis and remote hemorrhage were commonly seen inside the lesion, and vasculature with flow voids was commonly found inside or around the lesion. In addition, most of the lesions showed unclear boundary indicating invasiveness of the tumor. Mass effect was indicated by compressed ipsilateral brain parenchyma and ventricle and the shifted midline to the contralateral side. As expected, on the structural MRI, the glioma model showed MR characteristics similar to patient’s gliomas. The findings on heterogeneity of the GSC glioma model were consistent with previous findings, further demonstrating the feasibility and reliability of GSC xenografts as a glioma model.

On functional and metabolic imaging, most lesions showed an increase of intensity on DWI, similarly more than half of lesions showed an increase uptake of 18F-FDG under PET. After quantitative analysis of the tumor regions, significant negative correlation was found between the ADC and the SUV_max_ values. GSCs xenografts are tumors with high cellularity, making intercellular space very narrow. Therefore, due to the limitation of cell membrane, the diffusion of water molecules in the tumor is significantly restricted. The higher the cellularity is, the narrower the intercellular space is, so the more restrictive the diffusion of water molecules is and the lower the ADC value is. On the other side, the tumor cells need plenty of glucose as energy source when they divide and proliferate. Therefore, the higher the cellularity is, the more glucose the tumor requires, therefore the more 18F-FDG the tumor uptakes. That is the reason why there is a significant negative correlation between the ADC and the SUV_max_ value in the tumor region.

On the other hand, characteristics of molecular expression were also assessed using immunohistochemical staining in this study, including Ki-67, EGFR, VEGF, MGMT and HIF-1α. It was found that GSC tumor showed strong expression of Ki-67, moderate expression of EGFR and VEGF, and weak expression of MGMT and HIF-1α. As far as we know, the moderate to strong expression of Ki-67, EGFR and VEGF were well consistent with those of human GBM, leading to high cellularity and high angiogenesis in GBM. Of note, the Ki-67 expression in GSC tumor seemed to be a little higher than those of human gliomas, which may be due to the immune deficiency of the nude rats. Nude rat does not produce T cells, resulting in that cellular proliferation of the GSC tumor lacks of inhibition. In contrast, weak expression of MGMT indicates MGMT promoter is highly methylated, and weak expression of HIF-1α means low hypoxia in the GSC gliomas. However, subsequent validation is still required for MGMT and HIF-1α due to the weak expression in all tumor lesions.

In this study, no significant correlation was further observed between imaging features and most of the immunohistochemical features in this study with the exception of that between solid tumor size and EGFR. In other words, significant difference was merely observed in tumor size between tumor lesions with positive EGFR expression and those with negative expression, with larger size found in lesions with positive expression. In addition, tumor with positive VEGF expression seemed to have larger tumor size as well, and have higher probability to contain vasculature in or around the tumor. These findings well explained the effect of EGFR and VEGF expression on the growth and angiogenesis of the tumor.

This study complements the imaging and IHC characteristics of GSCs gliomas on the basis of existing studies, especially DWI, PET and IHC characteristics. On one hand, DWI and ^18^F FDG PET are important and representative functional or metabolic imaging methods in clinic, which can indicate the changes of tumor earlier than structural MRI. On the other hand, IHC characteristics are closely related to the biological behavior characteristics of tumors. These complementary characteristics may provide new insights to confirm the heterogeneity of GSCs gliomas.

This study also has limitations. The GSC model is made by implantation of GSC into the brain of the immuno-deficient rats, which does not produce T cells, but it has been demonstrated that T cells exist in gliomas and contribute to their natural history (25). Another shortcoming is that this study did not discuss the gadolinium based contrast-enhanced T1WI of GSC glioma model as previous studies did, because this study focused mainly on the characteristics of tumor based on DWI and PET, which would serve as a supplement or an improvement for characteristics of GSC glioma model. Although the ^11^C-MET PET is a more accurate imaging method than ^18^F FDG PET in assessing the gliomas, the ^18^F FDG were chosen because it is a more widely used imaging agent in clinical practice. In addition, the heterogeneity in multiple region of each tumor was not assessed in this study, which needs to be further studied.

In conclusion, the orthotopic xenograft tumor of GSCs showed significant heterogeneity and invasiveness on imaging, which well mimics the characteristics of human gliomas. In addition, strong expression of Ki-67, partial expression of EGFR and VEGF, and weak expression of MGMT and HIF-1α was oberserved on immunohistochemical staining, These supplementary characteristics will make GSC glioma model a better test platform for glioma.

## Data availability statement

The raw data supporting the conclusions of this article will be made available by the authors, without undue reservation.

## Ethics statement

The animal study was reviewed and approved by Animal Research Committee of the Tongji Medical College of Huazhong University of Science and Technology.

## Author contributions

All authors contributed to the study conception and design. Material preparation was by RJ, JuZ, YL, NS, and JJ. Data collection, and analysis were performed by RJ, JuZ, YL, JiZ, HZ, JJ, and WZ. The first draft of the manuscript was written by RJ, YL, and WZ. All authors contributed to the article and approved the submitted version.
